# Autonomic Dysregulation Mediates the Association Between Childhood Trauma and Pain Severity: Evidence from a Mediation Model

**DOI:** 10.3390/healthcare13182310

**Published:** 2025-09-16

**Authors:** Eleonora C. V. Costa, Patrícia Gonçalves, Fernando Martins, Sílvia Monteiro, Carla Pais-Vieira

**Affiliations:** 1 Centro de Estudos Filosóficos e Humanísticos, Faculdade de Filosofia e Ciências Sociais, Universidade Católica Portuguesa, 4710-297 Braga, Portugal; tichaa200@gmail.com; 2 North Regional Health Administration, 4000-477 Porto, Portugal; 3 Research and Support Center for Specific Victims, Portuguese Guarda Nacional Republicana, 1200-092 Lisbon, Portugal; martins.fmm@gnr.pt (F.M.); monteiro.srt@gnr.pt (S.M.); 4 Department of Education and Psychology, University of Aveiro, 3810-198 Aveiro, Portugal or cvieira@ucp.pt; 5 Centro de Investigação Interdisciplinar em Saúde (CIIS), Faculdade de Ciências da Saúde e Enfermagem (FCSE), Universidade Católica Portuguesa, 1649-023 Lisbon, Portugal

**Keywords:** childhood trauma, autonomic reactivity, pain severity, mediation analysis, integrated healthcare, trauma-informed care

## Abstract

**Background:** Childhood trauma is increasingly recognized as a key risk factor for autonomic nervous system (ANS) dysregulation and chronic pain. However, the mechanisms underlying this association remain insufficiently explored, particularly within integrated healthcare frameworks. **Objective:** This study examined whether autonomic reactivity mediates the relationship between childhood trauma and pain severity while accounting for age and gender. **Methods:** A total of 124 participants—64 with formally documented interpersonal trauma and 60 without—completed validated measures of childhood trauma (CTQ), cumulative trauma (LEC-17), autonomic reactivity (BPQ), and pain severity (BPI). Group comparisons, correlation analyses, and hierarchical regressions were used to assess associations among variables. A mediation model was used to test whether autonomic reactivity explained the trauma–pain relationship. **Results:** Trauma-exposed participants showed significantly higher autonomic reactivity than those without, while pain severity did not differ significantly between groups (*p* < 0.001). Childhood physical and emotional abuse was strongly associated with autonomic reactivity and moderately associated with pain severity but not directly linked to pain. Mediation analysis supported a full mediation, with childhood trauma predicting pain severity indirectly via autonomic reactivity (β = 0.220, 95% CI [0.087–0.422], *p* = 0.009). A preliminary gender effect on the trauma–ANS pathway was observed but was not sustained in weighted models correcting for sample imbalance. **Conclusions:** Autonomic dysregulation was found to mediate the link between childhood trauma and pain vulnerability. Incorporating autonomic assessment into trauma-informed, integrated healthcare could inform early detection and tailored interventions, with preliminary evidence suggesting generalizability across gender.

## 1. Introduction

Childhood trauma—including physical, emotional, or sexual abuse—is a well-established risk factor for long-term dysregulation of stress-responsive systems, notably the hypothalamic–pituitary–adrenal (HPA) axis and the autonomic nervous system (ANS). Trauma exposure often induces an early state of HPA hyper-responsivity, marked by exaggerated cortisol release, which can later shift toward blunted cortisol secretion and hypo-responsivity [1,2]. These alterations are reinforced by neuroplastic mechanisms, including stress-induced epigenetic modifications and remodeling of prefrontal–limbic circuits, which consolidate maladaptive stress responses over time [3,4]. Exposure to adversity during sensitive developmental windows has been linked to enduring structural and functional alterations in brain regions involved in affect regulation and executive control, such as the amygdala, hippocampus, and prefrontal cortex [5,6]. Together with increased allostatic load, these neurobiological alterations result in persistent dysregulation across multiple physiological systems [3,7,8]. Globally, chronic pain affects approximately 20–30% of adults, representing a major public health burden with substantial socioeconomic costs [9,10]. Importantly, rates are disproportionately higher among individuals with a history of childhood trauma, with epidemiological studies reporting 40–60% prevalence of chronic pain conditions in trauma-exposed populations, compared to 20–30% in the general population [11,12,13].

In parallel, early adversity is associated with autonomic dysregulation—characterized by heightened sympathetic tone, reduced parasympathetic activity, and lower heart rate variability [14,15]—as well as with chronic low-grade inflammation via neuroimmune alterations [7]. Polyvagal theory proposes that trauma disrupts ventral vagal regulatory pathways, shifting individuals into defensive physiological states that impair emotion regulation and visceral homeostasis [16,17]. Disruptions in interoceptive networks may also compromise bodily self-awareness and amplify somatic distress [17,18].

Clinically, trauma-exposed individuals show elevated rates of functional somatic syndromes—such as fibromyalgia, irritable bowel syndrome, and chronic pelvic pain—often linked to central sensitization and altered autonomic reactivity [19,20]. Longitudinal findings indicate that childhood trauma can predict greater pain severity and functional interference even years later [21]. Nonetheless, not all individuals exposed to early adversity develop chronic pain, and systematic reviews and meta-analyses point to substantial variability in the strength and consistency of trauma–pain associations. Earlier reviews reported robust associations between childhood maltreatment and chronic pain outcomes [13,22], whereas more recent evidence highlights heterogeneity across populations, pain conditions, and methodologies. For example, large-scale reviews confirm an elevated risk of chronic pain among trauma-exposed adults [23,24], while others emphasize modest or inconsistent effects once confounders are considered [25,26]. This heterogeneity suggests that the effect of trauma on pain may be indirect and mediated by physiological and psychological mechanisms rather than explained by a simple causal link.

Theoretical models such as the neurovisceral integration framework [16], polyvagal theory [16], and interoceptive predictive coding [27,28] converge on the idea that early adversity disrupts central systems involved in autonomic regulation, emotional modulation, and bodily awareness. These disruptions may impair top-down control over nociceptive input and contribute to heightened pain perception, even in the absence of ongoing tissue damage. Rather than acting as a direct cause of chronic pain, trauma may increase vulnerability through these intermediate pathways—particularly by shaping patterns of autonomic reactivity and interoceptive dysregulation [17,18].

Empirical support for this mechanism is emerging, but evidence remains fragmented. While autonomic dysregulation has been repeatedly implicated as a plausible mediator of trauma-related outcomes [16,29], most studies examine either trauma–autonomic links or autonomic–pain links in isolation. For example, Kolacz et al. (2020) found that autonomic reactivity partially mediated the association between childhood maltreatment and current PTSD and somatic symptoms [30]. In addition, Garland et al. (2019) and Liddell et al. (2016) demonstrated that reduced heart rate variability mediated trauma-related cognitive and emotional dysregulation [31,32]. Other studies on chronic pain samples report similar patterns [23,24], reinforcing the hypothesis that autonomic dysregulation may be a key mechanism linking trauma and pain. Nonetheless, very few investigations have directly tested whether autonomic dysregulation mediates the trauma–pain association in clinical or community populations. This represents the key empirical gap addressed by the present study. From a translational perspective, this mechanistic model justifies integrated care approaches that address both psychological and physiological aspects of trauma. Trauma-informed interventions targeting autonomic regulation—such as vagus nerve stimulation, paced breathing, or HRV biofeedback—have shown promise in reducing pain and emotional distress [33]. These strategies align with integrated healthcare models that aim to treat the individual holistically, particularly those with complex trauma histories [18].

To address this gap, the present study tested whether autonomic reactivity mediates the association between childhood trauma and pain severity in a combined sample with and without documented interpersonal trauma. We hypothesized that: (1) greater trauma exposure would be associated with increased autonomic reactivity; (2) autonomic reactivity would predict greater pain severity; and (3) the trauma–pain association would be fully mediated by autonomic dysregulation. Demonstrating this indirect pathway would support a reconceptualization of chronic pain as a trauma-related condition sustained by physiological dysregulation, with implications for integrated trauma-informed care.

## 2. Materials and Methods

### 2.1. Participants and Procedures

This study included 124 adult participants, all recruited from clinical healthcare settings between November 2022 and September 2023. To ensure sufficient variability in trauma exposure, participants were categorized according to the presence or absence of formally documented interpersonal trauma, rather than presumed clinical status.

The trauma-exposed group (n = 64) consisted of individuals referred by the Nucleus of Research and Support to Specific Victims (NRSSV) of the Portuguese police—a specialized service providing psychosocial and legal assistance to victims of interpersonal aggression. Eligibility for this group required having filed at least one formal criminal complaint involving physical, psychological, or sexual violence. The comparison group (n = 60) included participants recruited from public and private primary healthcare services in the same geographic region. These individuals met the same inclusion criteria—being 18 years or older, Portuguese-speaking, and capable of providing informed consent—but had no formally documented trauma exposure. Participants were not explicitly matched on age or socioeconomic status; however, recruitment from similar clinical settings in the same region was intended to reduce potential demographic disparities between groups. In addition, age and gender were statistically controlled in all analyses to minimize potential confounding.

Across the total sample, the mean age was 37.45 years (SD = 13.48; range = 18–84), and the majority of participants were female (66.9%). Gender distribution did not differ significantly between groups.

All participants completed a standardized 60 min assessment protocol that included a structured interview and self-report questionnaires. Assessments were conducted individually in private settings by trained graduate students in Clinical and Health Psychology, under close supervision from licensed psychologists, to ensure procedural consistency and minimize interviewer bias. Interviewers were not blinded to group allocation due to the nature of recruitment sources and the documentation required for eligibility; however, standardized procedures and close supervision were implemented to mitigate potential bias.

Study procedures were approved by the NRSSV Institutional Review Board and Ethics Committee. All participants provided written informed consent after receiving a detailed explanation of this study’s aims, methods, and ethical safeguards, including their right to withdraw at any time without penalty. No financial or material incentives were provided.

This grouping strategy was adopted to ensure the inclusion of participants with documented trauma histories and to enable a methodologically and ethically appropriate comparison across varying levels of trauma exposure. All primary analyses—including regression and mediation models—were conducted using the full sample (N = 124), treating trauma exposure as a continuous variable.

### 2.2. Self-Report Measures

#### 2.2.1. Sociodemographic and Clinical Data

A structured interview was conducted to collect sociodemographic information, including age, gender, and group allocation (trauma-exposed group vs. comparison group). These variables were used to characterize the sample and were examined as potential covariates in subsequent analyses.

#### 2.2.2. Childhood Trauma

Childhood trauma was assessed using an abbreviated 11-item version of the Childhood Trauma Questionnaire (CTQ; [34]), which evaluates adverse experiences prior to age 16. Two subscales were analyzed: (a) childhood physical and emotional abuse (CPEA; 5 items (e.g., “When I was 15 years old or younger, people in my family hit me so hard that it left me with bruises or marks”)) and (b) childhood sexual abuse (CSA; 6 items; e.g., “When I was 15 years old or younger, I believe that I was sexually abused”). Items were rated on a 5-point Likert scale (1 = never true to 5 = always true). Internal consistency in the present sample was acceptable for CPEA (α = 0.73) and excellent for CSA (α = 0.98). However, CSA responses showed a pronounced floor effect (92.7% scored at baseline), and this subscale was therefore excluded from inferential analyses.

#### 2.2.3. Cumulative Trauma Exposure

Lifetime trauma exposure was measured using the Life Events Checklist for DSM-IV (LEC-17 [35]; Portuguese version [36]), which assesses whether each of 17 potentially traumatic events (e.g., natural disasters, physical assault, and serious injury) was directly experienced, witnessed, or learned about. A cumulative score was computed by summing all endorsed events across exposure types, with higher values reflecting greater trauma burden. Internal consistency in this study was good (α = 0.88).

#### 2.2.4. Autonomic Reactivity

Autonomic symptoms were assessed using the autonomic reactivity domain of the Body Perception Questionnaire (BPQ; [37]; Portuguese version: [38]), which measures the frequency of stress-related sensations in autonomically innervated organs. Items are rated on a 5-point Likert scale (1 = never to 5 = always). The domain comprises two subscales—supradiaphragmatic reactivity (15 items) and subdiaphragmatic reactivity (6 items), with one overlapping item—and reflects general physiological reactivity. Internal consistency for the total BPQ autonomic reactivity score was excellent (α = 0.96). Although direct physiological measures (e.g., heart rate variability and electrodermal activity) were not collected, the BPQ-SF was used as a validated proxy of autonomic reactivity. The instrument demonstrates a robust factor structure and strong psychometric reliability [38,39]. Importantly, convergent validity has been established between BPQ scores and sensor-based physiological indices of autonomic functioning, including heart rate variability, wearable sensor data, and biometric markers [40,41,42]. More recent work further links BPQ responses to neurocardiac and interoceptive processes measured by EEG and HRV [43]. This evidence supports the use of the BPQ-SF as a valid self-report proxy of autonomic reactivity when direct physiological measures are not feasible.

#### 2.2.5. Pain Severity

Pain severity was assessed using the pain intensity scale of the Brief Pain Inventory (BPI [44]; Portuguese version [45]). The BPI includes ratings of worst, least, average, and current pain on a numerical scale ranging from 0 (no pain) to 10 (worst imaginable pain). The pain severity scale showed excellent psychometric properties across populations (α = 0.92–0.96 in this sample).

Together, these validated self-report instruments align with this study’s conceptual model and hypotheses, offering a comprehensive and reliable assessment of trauma exposure, autonomic reactivity, and pain severity across both participant groups.

### 2.3. Statistical Analysis

All statistical analyses were conducted using JASP (Version 0.19.3.0; JASP Team, Amsterdam, The Netherlands, 2023), with a two-tailed significance threshold set at *p* < 0.05. Prior to inferential testing, descriptive statistics were calculated for all study variables, including means, standard deviations, medians, interquartile ranges, and distribution indices (skewness and kurtosis). Normality assumptions were evaluated using the Shapiro–Wilk test and visual inspection of histograms and Q–Q plots. Variables with skewness < 2 and kurtosis < 7 were treated as approximately normally distributed and analyzed using parametric methods (CPEA, LEC, BPQ subscales, and pain severity).

Although both childhood physical and emotional abuse (CPEA) and childhood sexual abuse (CSA) were assessed, CSA exhibited an extreme floor effect, with 92.7% of participants scoring at the baseline value, resulting in negligible variability and pronounced non-normality (skewness > 4, kurtosis > 10). Consequently, CSA was reported only descriptively and was not included in group comparisons, correlations, regressions, or mediation models. The CTQ subscales assess conceptually distinct trauma domains: physical/emotional abuse (CPEA) and sexual abuse (CSA). Although these forms of adversity often co-occur in clinical and epidemiological studies [46,47], in the present sample, CSA showed very limited variability, precluding its inclusion in inferential analyses. Thus, CPEA was retained as the primary indicator of childhood trauma. This decision reflects the characteristics of our study population rather than an assumption of conceptual overlap, and it is consistent with prior evidence that different trauma types converge on common pathways of autonomic dysregulation and pain vulnerability [1,48].

Group differences between participants with and without documented interpersonal trauma were examined using independent sample *t*-tests for continuous variables. Effect sizes were reported as Cohen’s d and interpreted according to conventional thresholds (0.20 = small, 0.50 = medium, and 0.80 = large). For variables that violated normality or homogeneity of variances, nonparametric Mann–Whitney U tests were conducted to confirm the robustness of findings.

Additionally, the Life Events Checklist (LEC-17) was included as a measure of cumulative lifetime trauma exposure, providing a broader index of traumatic burden beyond childhood. Although not included in the mediation model, LEC-17 scores were used as an intermediate predictor in hierarchical regression models to examine whether trauma accumulation across the lifespan contributes to autonomic reactivity.

Bivariate correlations were computed to evaluate the associations between childhood trauma (CPEA), cumulative trauma exposure (LEC), autonomic reactivity (BPQ), pain severity, and sociodemographic covariates (age and gender). Pearson correlations were used for approximately normally distributed variables, whereas point-biserial correlations were applied for dichotomous variables such as gender. These correlations served both as descriptive indicators of the strength and direction of associations and as prerequisites for the hierarchical regression and mediation models.

Hierarchical linear regression analyses were then conducted to examine the incremental predictive effect of sociodemographic variables (gender and age) and trauma-related predictors (LEC-17 and CPEA) on autonomic reactivity (BPQ) and pain severity. The regression models were specified in sequential blocks to evaluate the change in explained variance (ΔR^2^) when adding predictors. Specifically, Block 1 included sociodemographic covariates (gender and age), Block 2 added cumulative trauma exposure (LEC-17), and Block 3 added childhood physical and emotional abuse (CPEA). For each model, R^2^ change, F-change, and model selection criteria (Akaike Information Criterion [AIC] and Bayesian Information Criterion [BIC]) were inspected. Multicollinearity was evaluated using Variance Inflation Factor (VIF) and tolerance values, with VIF < 5 and tolerance > 0.20 considered acceptable [49]. The Durbin–Watson statistic was also computed to assess residual autocorrelation. Regression coefficients were reported as both unstandardized (B) and standardized (β) estimates, with their respective standard errors, t-values, and significance levels. Additionally, part and partial correlations were calculated to assess the unique contribution of each predictor.

Notably, cumulative trauma exposure (LEC-17) explained additional variance in autonomic reactivity beyond age and gender, suggesting that lifetime trauma burden contributes to heightened physiological reactivity. However, given this study’s primary focus on childhood trauma, LEC-17 was treated as a secondary predictor and was not integrated into the mediation model. This is consistent with evidence that, although cumulative trauma can increase physiological vulnerability through allostatic load [50], early-life trauma represents a particularly sensitive period for shaping long-term autonomic dysregulation [51].

Finally, mediation analyses were performed to test whether autonomic reactivity (BPQ) mediated the association between childhood trauma (CPEA) and pain severity. Given the hypothesized absence of a direct association between CPEA and pain severity, a full mediation model was expected. Mediation models were estimated using maximum likelihood (ML) with 5000 bias-corrected bootstrap resamples to obtain robust confidence intervals for indirect effects, as recommended for small-to-moderate sample sizes [52]. Model fit indices (χ^2^, CFI, TLI, RMSEA, and SRMR) supported the adequacy of the mediation model, with all parameters falling within acceptable thresholds. Statistical power for detecting indirect effects in the mediation model was also estimated following Fritz and MacKinnon (2007) [53]. For the present sample size (N = 124), this study was sufficiently powered to detect moderate mediation effects. To examine whether the mediation model was invariant across gender, two additional analyses were conducted using structural equation modeling in R (lavaan package, v. 0.6-19). First, a multigroup mediation model was estimated to assess potential differences in path coefficients between male and female participants. A score test for parameter invariance was performed to identify whether any individual paths significantly differed across groups. Second, given the unequal sample sizes by gender (83 women and 41 men), a weighted mediation model was computed using inverse-probability sampling weights. This approach equalized the statistical contribution of each group and allowed the estimation of group-specific mediation paths while accounting for sample imbalance. Both models included gender as a grouping variable, and sampling weights were derived based on the ratio of group sizes. These additional analyses were designed to verify the robustness of the mediation findings across gender and to address potential biases associated with unequal group representation. All variables were checked for missing data, and no missing values were identified; therefore, all analyses were conducted on complete cases.

This analytical strategy was designed to sequentially test the study hypotheses: (1) characterize group differences in trauma exposure, autonomic reactivity, and pain severity; (2) establish bivariate relationships between key variables; (3) evaluate the incremental contribution of childhood trauma and autonomic reactivity to pain severity beyond sociodemographic factors; and (4) test the hypothesized indirect effect of childhood trauma on pain severity through autonomic reactivity.

## 3. Results

### 3.1. Descriptive Statistics and Group Comparisons

The total sample consisted of 124 participants (M_age = 37.45, SD = 13.48; range = 17–84), of whom 66.9% were women. Participants were classified into two groups based on confirmed interpersonal trauma exposure: the trauma-exposed group (n = 64) and the comparison group (n = 60), both recruited from healthcare settings. Group characteristics are detailed in Table 1.

Childhood physical and emotional abuse (CPEA) was the most prevalent form of early adversity, with an overall mean score of 7.96 (SD = 3.49; range = 0–25). Although moderately skewed (skewness = 1.99) and leptokurtic (kurtosis = 5.24), the distribution met acceptable thresholds for parametric testing. The trauma-exposed group reported significantly higher CPEA scores (M = 8.88, SD = 4.13) than the comparison group (M = 6.99, SD = 2.30), with t(122) = −3.12, *p* = 0.002, and Cohen’s d = 0.56.

Childhood sexual abuse (CSA) was less frequent, with a mean of 6.88 (SD = 3.92) and a high floor effect (skewness = 4.84; kurtosis = 23.26), indicating very limited variability. Given the low prevalence and non-normal distribution, CSA was reported descriptively and excluded from inferential analyses.

Cumulative exposure to potentially traumatic events across the lifespan (LEC-17) showed a mean of 10.41 (SD = 8.10), with mild skewness (0.77) and acceptable distributional properties. As expected, the trauma-exposed group reported significantly higher scores (M = 13.20, SD = 8.43) than the comparison group (M = 7.43, SD = 6.60), with t(122) = −4.23, *p* < 0.001, and d = 0.76.

Autonomic reactivity (BPQ) was also significantly higher in the trauma-exposed group (M = 38.19, SD = 18.26) compared to the comparison group (M = 29.28, SD = 7.13), with t(122) = −3.53, *p* < 0.001, and Cohen’s d = 0.64, despite deviations from normality (Shapiro–Wilk *p* < 0.001). This result was corroborated by the Mann–Whitney test (U = 1467.50, *p* = 0.023). Pain severity, assessed with the Brief Pain Inventory (BPI), showed an overall mean of 7.69 (SD = 8.58) on the summed 0–40 scale. When expressed in the conventional 0–10 item format, this corresponds to an average of 1.92, indicating low-to-moderate pain severity in the sample. No significant group differences were observed between trauma-exposed (M = 7.03, SD = 9.36) and comparison participants (M = 8.40, SD = 7.68), with t(122) = 0.89, *p* = 0.377, and d = 0.16. Overall pain severity scores were low to moderate and showed wide variability (M = 7.69, SD = 8.58; range 0–40), which likely attenuated between-group mean differences despite robust differences in autonomic reactivity. This supports the hypothesis that pain severity may be modulated by intermediate mechanisms such as autonomic dysregulation rather than being directly influenced by trauma exposure per se.

Overall, the data distributions for CPEA, LEC-17, BPQ, and pain severity allowed for parametric analyses, whereas CSA was excluded due to extreme skewness. There were no missing data in the dataset.

### 3.2. Bivariate Correlations

The results of the bivariate correlations are summarized in Table 2. These analyses examined the associations among childhood physical and emotional abuse (CPEA), total autonomic reactivity (BPQ), pain severity, and potential covariates (age and gender). Correlations were computed with 95% confidence intervals, which did not alter the pattern of statistical significance (see Table 2 note). Given the significant deviation from multivariate normality (Shapiro–Wilk W = 0.882, *p* < 0.001), both Pearson’s r and Spearman’s ρ were computed to ensure robustness.

As shown in Table 2, CPEA was moderately and positively correlated with autonomic reactivity (Pearson’s *r* = 0.524, *p* < 0.001; Spearman’s *ρ* = 0.455, *p* < 0.001), indicating that greater exposure to childhood trauma was associated with higher autonomic dysregulation. Autonomic reactivity was also significantly associated with pain severity (Pearson’s *r* = 0.408, *p* < 0.001; Spearman’s *ρ* = 0.369, *p* < 0.001), suggesting that individuals with higher autonomic dysregulation tended to report more severe pain.

In contrast, the correlation between CPEA and pain severity was not statistically significant (Pearson’s *r* = 0.120, *p* = 0.185; Spearman’s *ρ* = 0.034, *p* = 0.709), supporting the hypothesis of an indirect pathway from trauma to pain via autonomic reactivity.

Regarding demographic covariates, age showed a weak positive correlation with autonomic reactivity (Pearson’s *r* = 0.206, *p* = 0.022; Spearman’s *ρ* = 0.171, *p* = 0.041) but was not significantly related to pain severity. Gender (coded as 0 = female, 1 = male) was negatively associated with both autonomic reactivity (*r* = −0.194, *p* = 0.031) and pain severity (*r* = −0.208, *p* = 0.021), indicating that women reported slightly higher levels of both.

Together, these findings support the inclusion of autonomic reactivity as a mediator in the trauma–pain relationship and justify the retention of age and gender as covariates in subsequent regression and mediation models.

### 3.3. Hierarchical Regression Results

To examine the hypothesized mediation pathway linking childhood trauma to pain severity via autonomic reactivity, a series of hierarchical regression models was estimated (see Table 3).

#### 3.3.1. Step 1: Predicting Autonomic Reactivity (BPQ)

In the first step, total autonomic reactivity (BPQ) was regressed on demographic and trauma-related predictors. The baseline model (M_0_), which included age and gender, accounted for 8% of the variance in BPQ scores (R^2^ = 0.080, *p* = 0.006). In this model, age emerged as a significant predictor (β = 0.206, *p* = 0.020), with older participants reporting higher autonomic reactivity, while gender showed a marginal trend (β = −0.194, *p* = 0.028), suggesting that women (coded as 0) exhibited higher BPQ scores than men (coded as 1). In Model 1, the inclusion of cumulative trauma exposure (LEC-17) significantly improved model fit (ΔR^2^ = 0.213, *p* < 0.001), with LEC-17 predicting BPQ scores independently of demographics (β = 0.468, *p* < 0.001). Finally, Model 2 added childhood trauma (CPEA), which accounted for an additional 11.6% of variance in BPQ scores (ΔR^2^ = 0.116, *p* < 0.001). CPEA emerged as a robust predictor of autonomic reactivity (β = 0.370, *p* < 0.001), even after controlling for age, gender, and lifetime trauma. This pattern supports the first precondition for mediation: a significant association between the independent variable (CPEA) and the proposed mediator (BPQ).

#### 3.3.2. Step 2: Predicting Pain Severity from CPEA

To assess whether CPEA had a direct effect on pain severity, two models were compared. In the baseline model (M_0_), gender significantly predicted pain severity (β = −0.208, *p* = 0.021), with women reporting higher pain levels. However, the addition of CPEA in Model 1 did not significantly improve the model (ΔR^2^ = 0.010, *p* = 0.267), and CPEA itself was not a significant predictor (β = 0.115, *p* = 0.267). This absence of a direct effect is consistent with the assumption of full mediation, whereby the influence of childhood trauma on pain severity may be transmitted through other mechanisms, rather than exerted directly.

#### 3.3.3. Step 3: Predicting Pain Severity from Autonomic Reactivity

In a separate analysis, BPQ was added as a predictor of pain severity (Model 1), alongside gender. The inclusion of BPQ significantly increased the explained variance from 4.3% to 18.3% (ΔR^2^ = 0.140, *p* < 0.001). Autonomic reactivity was a strong predictor of pain (β = 0.382, *p* < 0.001), while gender was no longer significant in the presence of BPQ (β = −0.134, *p* = 0.113). This supports the second precondition for mediation, demonstrating that the mediator (BPQ) is significantly associated with the outcome (pain severity).

Across all models, multicollinearity was ruled out, with Variance Inflation Factor (VIF) values below 1.2 and tolerance above 0.84. Normality tests (Shapiro–Wilk, *p* < 0.001) indicated deviations from normality; however, the sample size (N = 124) supports the robustness of regression results under mild non-normality [54]. 

The hierarchical regression results satisfy the main conditions required for testing a mediation model: (1) CPEA significantly predicts autonomic reactivity (BPQ); (2) BPQ significantly predicts pain severity; and (3) CPEA does not directly predict pain severity when BPQ is included. Therefore, a mediation analysis was conducted to test the indirect effect of childhood trauma on pain severity via autonomic dysregulation. Full mediation was expected and tested using a bootstrapped approach with 5000 resamples [52,55].

### 3.4. Mediation Analysis

**Figure 1:**
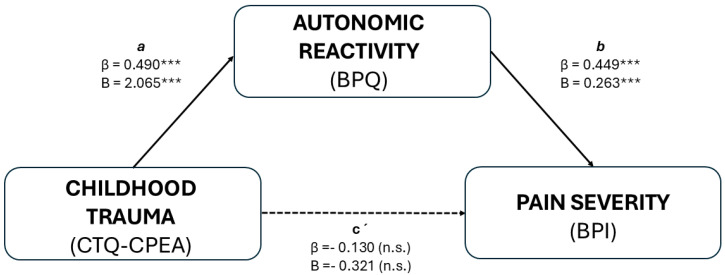
Mediation model testing whether autonomic reactivity mediates the association between childhood trauma and pain severity. Standardized (β) and unstandardized (B) path coefficients are shown for each effect. Path a represents the effect of childhood trauma (CTQ-CPEA) on autonomic reactivity (BPQ); path b reflects the effect of autonomic reactivity on pain severity (BPI); and path c′ represents the direct effect of childhood trauma on pain severity after accounting for the mediator. The indirect effect (a × b) was statistically significant, indicating a mediating role of autonomic reactivity in the association between childhood trauma and pain severity. Covariates (gender and age) were included in the model but are not depicted in the figure for simplicity. *p* < 0.001 ***.

As detailed in Table 3, the hierarchical regressions first established the necessary preconditions for mediation, namely that childhood trauma predicted autonomic reactivity (path a) and that autonomic reactivity predicted pain severity (path b), while the direct trauma–pain effect was non-significant (path c’). These preliminary results are conceptually summarized in the mediation model (Figure 1) and formally tested in the mediation analysis (Table 4). In the initial mediation model, which included gender and age as covariates, childhood trauma (CTQ_CPEA) significantly predicted autonomic reactivity (BPQ_AUTONOMIC_REACTIVITY; β = 0.490, *p* < 0.001), which, in turn, significantly predicted pain severity (PAIN_SEVERITY_SCALE; β = 0.449, *p* < 0.001). The indirect effect of childhood trauma on pain severity through autonomic reactivity was statistically significant (β = 0.220, *p* = 0.009), whereas the direct effect was not (β = −0.130, *p* = 0.313), indicating a full mediation pathway. Based on standard benchmarks for mediation magnitude [53], this corresponds to a moderate-to-large effect, indicating that autonomic reactivity accounts for a substantial proportion of the trauma–pain association. From a clinical perspective, this suggests that interventions targeting autonomic regulation may hold meaningful potential for mitigating trauma-related pain outcomes.

Power analysis indicated that with N = 124, this study had approximately 74% power to detect the observed indirect effect (β = 0.220, SE = 0.084, *p* = 0.009, 95% CI [0.087, 0.422]).

To assess whether the mediation model varied by gender, a multigroup structural equation model (SEM) was conducted. The global score test for parameter invariance was non-significant (χ^2^(3) = 4.388, *p* = 0.223), suggesting overall equivalence of the model across genders. However, as shown in Table 5, the univariate score test revealed a marginally significant difference for path a (CTQ_CPEA → BPQ; χ^2^(1) = 3.880, *p* = 0.049), indicating a potential gender-specific variation in how childhood trauma relates to autonomic reactivity.

To address the imbalance in group sizes (83 women vs. 41 men), a second SEM was estimated using inverse-probability sampling weights. This approach allowed for equal statistical contribution of each gender and more accurate estimation of group-specific mediation paths. As presented in Table 6, the weighted model replicated the main mediation pattern in both subgroups. The trauma → reactivity path (a) remained significant for both men (β = 0.427, *p* < 0.001) and women (β = 0.523, *p* < 0.001). The path from autonomic reactivity to pain (b) was also significant in both groups (men: β = 0.391, *p* < 0.001; women: β = 0.470, *p* < 0.001), while the direct path from trauma to pain (c) remained non-significant in both (men: β = −0.098, *p* = 0.315; women: β = −0.144, *p* = 0.315).

Taken together, these findings suggest that the mediation mechanism linking childhood trauma to pain severity via autonomic reactivity is robust and consistent across genders. Although a marginal gender difference emerged in the strength of the trauma–reactivity association, the overall mediation structure appears stable. This reinforces the generalizability of the proposed model and highlights the central role of autonomic dysregulation as a transdiagnostic pathway between early trauma and pain.

## 4. Discussion

This study examined whether autonomic dysregulation mediates the association between childhood physical and emotional abuse (CPEA) and pain severity in a mixed trauma-exposed and comparison sample. The analyses supported a full mediation structure, showing that the association between early trauma and pain severity operated indirectly via autonomic reactivity. Notably, the direct effect of trauma on pain became non-significant when autonomic dysregulation was accounted for, suggesting that psychophysiological processes—rather than direct somatic consequences—may explain how early adversity contributes to chronic pain risk. These results are consistent with theoretical and empirical frameworks that position autonomic reactivity as a key transdiagnostic mechanism linking trauma to somatic and emotional health outcomes [17,29]. The neurovisceral integration model [29], polyvagal theory [16], and interoceptive predictive coding models [28] all converge on the idea that early adversity disrupts central autonomic regulation, compromising bodily awareness, emotional modulation, and top-down control of physiological responses. These alterations may amplify pain sensitivity, even in the absence of tissue damage. Empirical findings reinforce this view. For example, reduced heart rate variability (HRV)—a marker of impaired parasympathetic tone—has been repeatedly associated with both trauma exposure and chronic pain [56,57]. Recent reviews also highlight the heterogeneity of trauma–pain associations, suggesting that intermediate physiological mechanisms, such as autonomic dysregulation, are critical in explaining why not all individuals exposed to early adversity develop pain-related conditions [23,24,25].

Importantly, this mediated pathway was identified in a heterogeneous nonclinical sample, underscoring the broad applicability of trauma-informed approaches beyond psychiatric populations. These findings align with increasing advocacy for integrated healthcare models that address the biopsychosocial complexity of pain, trauma, and physiological dysregulation [58,59]. Incorporating autonomic markers into routine assessment may facilitate early identification of at-risk individuals and support the development of precision interventions tailored to individual psychophysiological profiles.

### 4.1. Childhood Trauma and Autonomic Dysregulation

The significant association observed between childhood physical and emotional abuse (CPEA) and heightened autonomic dysregulation is consistent with a broad literature base identifying the autonomic nervous system (ANS) as a critical mediator of the biopsychosocial impact of early adversity. According to polyvagal theory, early-life trauma compromises the development of the ventral vagal complex, which governs social engagement and visceral regulation, leading to a shift toward defensive autonomic states—namely, sympathetic hyperarousal (“fight or flight”) or parasympathetic shutdown (“freeze”) [16,17]. These alterations reduce autonomic flexibility and hinder physiological adaptation to environmental demands.

Empirical findings support this theoretical framework. Individuals with histories of childhood maltreatment often display reduced vagally mediated heart rate variability (vmHRV)—a biomarker of parasympathetic tone and self-regulatory capacity [14,15]. A recent meta-analysis by Sigrist et al. (2021) [60] quantitatively assessed the effect of early-life maltreatment on resting HRV, finding a significant association that varied as a function of age and psychopathology and supporting a medium-to-small effect size for decreased parasympathetic tone in trauma-exposed groups. Lower HRV has been repeatedly linked to impaired emotion regulation, elevated stress reactivity, and increased risk of somatic and psychiatric comorbidities [60].

Moreover, trauma-related autonomic alterations extend beyond cardiovascular parameters. Kolacz and Porges (2018) found that trauma-exposed individuals report elevated autonomic symptoms across gastrointestinal, respiratory, and cardiovascular domains, reflecting heightened interoceptive sensitivity and impaired homeostasis [17]. These findings support the idea that the ANS serves as a transdiagnostic conduit through which early adversity shapes diverse health outcomes.

Cumulative trauma exposure—commonly assessed through instruments such as the Life Events Checklist (LEC)—has been proposed to contribute to chronic autonomic dysregulation, in line with the theory of allostatic load [50]. Supporting this hypothesis, longitudinal evidence from Jeon et al. (2024) showed that individuals hospitalized for physical injuries who also reported a history of childhood abuse exhibited sustained alterations in autonomic balance, reflected in low- and high-frequency heart rate variability (HRV) indices that significantly predicted increased risk for post-traumatic stress disorder (PTSD) at a two-year follow-up [61]. In contrast, no such association was found among those without prior abuse exposure. These findings highlight how cumulative adversity may produce lasting imbalances in sympathetic–parasympathetic regulation, thereby increasing vulnerability not only to psychiatric outcomes such as PTSD and depression but also to chronic somatic conditions, including pain and systemic inflammation.

Taken together, the present findings contribute to a growing body of evidence identifying the autonomic nervous system (ANS) as a physiological interface between adverse childhood experiences and long-term health trajectories [17,62]. Disruptions in autonomic regulation have been linked to a range of conditions—beyond mental health—including fibromyalgia, irritable bowel syndrome, and functional somatic syndromes [25,57]. As such, psychophysiological measures—such as heart rate variability monitoring or self-report tools like the Body Perception Questionnaire (BPQ)—may offer accessible screening strategies to identify autonomic dysregulation in both specialized and primary care settings (e.g., [58]). Their inclusion in integrated trauma-informed healthcare could enhance early detection and treatment of stress-related health vulnerabilities.

### 4.2. Autonomic Dysregulation and Pain Severity

The present findings demonstrate a significant association between heightened autonomic reactivity and increased pain severity, reinforcing the role of autonomic nervous system (ANS) dysfunction in the pathophysiology of chronic pain. This relationship is consistent with a growing body of literature indicating that sympathetic overactivation and reduced parasympathetic modulation can exacerbate pain through neurophysiological and affective pathways (e.g., [29,50]).

According to the neurovisceral integration model, the prefrontal cortex exerts regulatory control over subcortical structures involved in both autonomic output and nociceptive processing. Disruptions in this network—common in trauma-exposed or chronically stressed individuals—can reduce vagal tone, leading to hypervigilance, impaired emotion regulation, and decreased top-down modulation of pain [29,63]. Reduced heart rate variability (HRV), a biomarker of diminished parasympathetic flexibility, has consistently been linked to increased pain sensitivity, lower pain thresholds, and impaired habituation to repeated painful stimuli [57].

Autonomic dysregulation may also interfere with the brain’s processing of bodily signals through dysfunctional interoceptive prediction. Within predictive coding frameworks, pain is not only a response to nociceptive input but also a product of top-down inferences about bodily threat [27,28]. In this view, autonomic hyperreactivity may generate excessive or noisy interoceptive input, which is then misinterpreted as painful due to failures in prediction error minimization—particularly in individuals with trauma histories or emotional dysregulation [28,64].

Clinical and experimental evidence further supports the role of ANS dysfunction in pain states. For example, individuals with fibromyalgia and related functional somatic syndromes (such as irritable bowel syndrome and chronic fatigue syndrome) frequently exhibit a predominant sympathetic tone and reduced baroreflex sensitivity, both of which correlate with increased pain severity and impaired autonomic recovery [65,66]. A meta-analysis by Tracy et al. (2016) further confirmed that lower heart rate variability (HRV) is consistently associated with greater pain intensity across both clinical and nonclinical populations [57].

These findings collectively support the conceptualization of autonomic dysregulation as a transdiagnostic vulnerability marker for chronic pain and related disorders. They also provide a mechanistic foundation for interventions aimed at restoring autonomic balance. This view is consistent with studies showing that pain severity in complex conditions is closely associated with psychological factors, underscoring the need to address both physiological and cognitive–affective mechanisms in clinical care [67]. Approaches such as vagus nerve stimulation, paced breathing, mindfulness-based stress reduction, and HRV biofeedback have shown promising results in improving autonomic flexibility and reducing pain severity [68,69]. Integrating such strategies into trauma-informed care models may enhance clinical outcomes by targeting the physiological substrates of pain perception.

#### Central Nervous System Connectivity and Pain Regulation

Beyond peripheral autonomic mechanisms, recent neuroimaging research highlights the role of large-scale brain network connectivity in pain and autonomic regulation. Chronic pain conditions have been associated with altered connectivity between the salience network (SN) and the default mode network (DMN), suggesting a neural basis for heightened pain sensitivity and impaired switching between interoceptive and exteroceptive states [70]. Dynamic fluctuations within the SN and pontine networks have also been shown to predict variations in moment-to-moment pain intensity in patients with chronic back pain and migraine [71]. Moreover, connectivity between the dorsal anterior cingulate cortex (dACC), periaqueductal gray (PAG), dorsolateral prefrontal cortex, and anterior insula has been directly linked to autonomic output during pain processing, as indexed by low-frequency heart rate variability (LF-HRV) [72]. Together, these findings suggest that trauma-related pain vulnerability may emerge from an interplay between autonomic dysregulation and maladaptive central connectivity in brain circuits governing stress and nociception.

### 4.3. Mediation Pathway: Trauma, Autonomic Dysregulation, and Pain

These findings align with theoretical models proposing that autonomic dysregulation may represent a psychophysiological pathway between childhood trauma and pain severity. The absence of a significant direct group effect on pain severity, despite higher autonomic reactivity in trauma-exposed participants, is consistent with an indirect-only mediation pattern [52,73]. This dissociation suggests that trauma operates as a latent vulnerability factor: it sensitizes central autonomic regulation, but pain manifestation depends on additional moderators, such as current nociceptive input, affective factors, and sleep or inflammatory processes. Thus, trauma may increase autonomic dysregulation broadly, while clinical pain expression varies across individuals, strengthening the rationale for testing mediation rather than direct group effects. This pattern is consistent with latent vulnerability models of childhood trauma, which propose that early adversity does not always translate into overt psychopathology or pain but leaves enduring alterations in stress-regulatory systems that bias responses to later challenges [74]. In this view, trauma-exposed individuals may carry a “physiological scar” in the form of heightened autonomic reactivity, while the emergence of clinical pain depends on additional contextual moderators, such as ongoing nociceptive input, inflammation, or affective dysregulation. Our findings therefore reinforce the idea that trauma confers vulnerability indirectly, through latent psychophysiological alterations, rather than exerting a uniform direct effect on pain outcomes. Notably, the absence of a significant direct effect between trauma and pain severity aligns with current transdiagnostic models positing that trauma does not invariably lead to chronic pain but exerts its influence via intermediary systems such as the autonomic nervous system (ANS) [23,24,25]. This finding helps clarify inconsistencies in the literature and supports the conceptualization of autonomic imbalance as a “silent mediator” that may operate independently of explicit trauma recall or clinical diagnosis [75,76].

This pathway is consistent with the neurovisceral integration model [29], which suggests that disruption in prefrontal–autonomic circuits compromises both emotional regulation and somatic control, thereby increasing vulnerability to pain. Additionally, interoceptive predictive coding theories propose that early adversity alters the brain’s interpretation of internal bodily signals; autonomic dysregulation may distort these signals, leading to amplified pain perception, even in the absence of ongoing nociceptive input [27,28]. This pathway is also consistent with interoceptive predictive coding frameworks, which conceptualize pain as a product of top-down predictions interacting with bottom-up bodily signals. In this view, autonomic hyperreactivity may generate noisy or excessive interoceptive input, increasing prediction errors and leading to maladaptive inferences of bodily threat [28,77]. Trauma-related alterations in autonomic regulation may therefore bias interoceptive priors toward hypervigilance, amplifying pain perception, even in the absence of nociceptive input. These mechanisms complement neurovisceral integration and polyvagal models, situating autonomic dysregulation within broader theories of brain–body predictive coding and highlighting its role as a mediator between early adversity and chronic pain.

Empirical studies lend further credibility to this mechanistic explanation. For example, Häuser et al. (2011) demonstrated that autonomic symptoms mediated the relationship between childhood maltreatment and symptom severity in fibromyalgia, even after adjusting for depressive symptoms [11]. In the context of irritable bowel syndrome, early adversity has been linked to heightened visceral pain sensitivity through altered vagal and limbic–autonomic pathways, a relationship that persists regardless of PTSD status [75,76]. These findings indicate that trauma-related dysregulation can remain latent and physiologically embedded, affecting individuals without overt psychological diagnoses.

Complementary case-based evidence describes altered pain perception in a trauma-exposed young adult with Riley–Day syndrome, illustrating how trauma history may interact with rare neurophysiological conditions to amplify pain vulnerability [78]. Importantly, our sample included individuals with self-reported trauma histories as well as clinical patients recruited from primary care settings, enhancing the ecological validity and relevance of the findings for real-world healthcare contexts. The mediation observed thus suggests that subclinical or unreported trauma may contribute to chronic pain via autonomic mechanisms that are not captured through self-report alone [79]. This highlights the need for screening tools that can detect underlying autonomic dysfunction—such as heart rate variability (HRV) or validated self-report instruments like the Body Perception Questionnaire [39].

These findings also have clinical implications. Non-invasive interventions, such as transcutaneous vagus nerve stimulation (tVNS), have shown promising effects on autonomic regulation. Studies have demonstrated that tVNS can increase heart rate variability (HRV) and reduce sympathetic activity, indicating a shift toward parasympathetic dominance [80,81,82]. Incorporating such interventions into trauma-informed, integrated healthcare models may enhance therapeutic outcomes by directly targeting the neurophysiological pathways mediating trauma-related somatic symptoms (e.g., [83]).

Overall, the absence of a direct trauma–pain link in this study does not contradict prior findings but rather supports a more nuanced understanding of trauma as a latent vulnerability factor, whose effects are mediated through dysregulated physiological systems. Recognizing this mediation pathway is essential for advancing precision interventions and improving care for individuals with trauma-related pain conditions.

### 4.4. Gender Effects and Structural Robustness

This study examined whether the mediating role of autonomic dysregulation in the association between childhood trauma and pain severity differed by gender. Although preliminary descriptive analyses revealed small differences in both autonomic reactivity and pain severity between men and women, these were not statistically significant. When the mediation model was tested using multigroup structural equation modeling with group-specific weights to account for sample imbalance, the indirect pathway—linking trauma to pain via autonomic dysregulation—remained invariant across gender. This structural robustness suggests that while gender may influence symptom intensity, the underlying psychophysiological mechanism remains consistent across sexes.

These results are consistent with the existing literature showing that trauma-related autonomic and pain dysregulation is evident in both men and women. For instance, Fillingim et al. (2009) noted that although women tend to report greater pain sensitivity and higher prevalence of chronic pain, these differences often reflect sociocultural, hormonal, and psychological factors rather than fundamental divergences in nociceptive pathways [84]. Similarly, experimental pain studies have shown that while women often report higher pain sensitivity, gender differences in conditioned pain modulation (CPM) diminish or disappear when psychological variables such as catastrophizing and anxiety are statistically controlled [84,85]. Moreover, a study using CPM found no significant differences between young men and women, suggesting comparable endogenous pain inhibitory capacity when confounders are accounted for [86].

With respect to autonomic functioning, Koenig and Thayer (2016) conducted a systematic review showing that trauma exposure is associated with reductions in heart rate variability (HRV) in both sexes, although baseline HRV may differ due to hormonal and developmental factors [63]. These findings support the notion that trauma exerts a pervasive dysregulating effect on the autonomic nervous system, transcending biological sex differences. Importantly, the preservation of the trauma–autonomic–pain mediation pathway across genders underscores the generalizability and clinical relevance of the proposed model. It suggests that autonomic dysregulation functions as a transdiagnostic vulnerability marker regardless of sex, highlighting the need for trauma-informed interventions that address physiological regulation in both men and women. This is particularly critical given evidence that men may underreport trauma due to stigma or adherence to traditional gender roles, leading to an underrecognition of trauma-related somatic symptoms in male patients [87].

Taken together, these findings support an inclusive gender-informed approach to trauma and pain assessment that integrates autonomic markers such as HRV and self-report measures across diverse healthcare settings.

### 4.5. Theoretical and Clinical Implications

The current findings strengthen a biopsychosocial understanding of chronic pain, identifying autonomic dysregulation as a physiological mechanism linking early-life trauma to long-term somatic vulnerability. This perspective moves beyond reductionist models that attribute chronic pain solely to peripheral nociceptive input or structural injury and instead incorporates neurophysiological, psychological, and contextual factors—consistent with integrative pain models developed over the past two decades [88,89,90]. Recent calls in the osteoarthritis field similarly emphasize the importance of investigating central pain mechanisms and tailoring prevention strategies to individual vulnerability profiles [91]. Such perspectives reinforce the need for research that bridges mechanistic insights with personalized translational approaches to pain management.

By demonstrating that autonomic reactivity mediates the association between childhood trauma and pain severity, our study supports the inclusion of autonomic markers as early indicators of health risk in trauma-exposed individuals—even in the absence of psychiatric diagnoses. This aligns with calls for trauma-informed care frameworks that incorporate both psychological and physiological dimensions of stress reactivity and regulatory function [92,93,94].

From a translational perspective, clinical integration of psychophysiological screening tools such as heart rate variability (HRV), skin conductance response, and self-report measures like the Body Perception Questionnaire (BPQ) may allow for earlier identification of patients with dysautonomia-linked pain, fatigue, or functional somatic syndromes [15,69]. Autonomic profiles may also guide personalized treatment strategies and support risk stratification in both primary care and mental health settings [95].

The clinical implications are equally notable. Interventions designed to restore autonomic balance—such as paced breathing, mindfulness-based stress reduction (MBSR), vagus nerve stimulation (VNS), and HRV biofeedback—have demonstrated promising results in reducing pain-related distress and improving affect regulation in trauma-exposed populations [96,97]. Integrating such strategies into trauma-informed care models may enhance clinical outcomes by targeting the physiological substrates of pain perception. Indeed, non-invasive strategies targeting autonomic regulation have shown efficacy in lowering physiological arousal and pain severity across both experimental and clinical contexts [98,99,100,101]. Somatic approaches, including trauma-informed yoga, sensorimotor psychotherapy, and body awareness practices, can also support autonomic regulation and foster embodiment in individuals with trauma-related dissociation, somatosensory hypervigilance, or dysregulated interoception [102,103]. Beyond the ANS, recent evidence implicates gut microbiome alterations in chronic pain, with lifestyle-based interventions showing potential to modulate these pathways, reinforcing the value of integrative and multidisciplinary approaches [104].

Furthermore, these interventions may enhance the efficacy of conventional trauma psychotherapies—such as trauma-focused cognitive behavioral therapy (CBT) or eye movement desensitization and reprocessing (EMDR)—by targeting physiological dysregulation that often underlies persistent somatic symptoms [94]. The inclusion of interoceptive training and autonomic modulation may thus bridge the historic divide between mind and body in trauma treatment [93,105].

In practical terms, autonomic assessment can be incorporated into healthcare pathways through both physiological and self-report tools. Heart rate variability (HRV), for instance, can be monitored non-invasively via portable sensors or wearable devices during routine clinical visits, providing real-time indicators of autonomic balance. Similarly, the Body Perception Questionnaire (BPQ) offers a brief validated self-report measure that can be administered in primary care or mental health settings to screen for dysautonomia-linked risk profiles. Together, these complementary approaches may enable earlier detection of trauma-related physiological vulnerability and inform trauma-informed referral and intervention strategies.

Overall, our findings underscore the relevance of autonomic dysregulation not only as a mechanism of symptom maintenance but also as a modifiable treatment target. Integrating autonomic assessment and regulation into trauma-informed care models offers a promising pathway to improving outcomes for individuals with chronic pain, functional syndromes, and complex trauma histories. Beyond clinical applications, the present findings also have clear preventive implications. The mediation pathway was identified in a heterogeneous and partly nonclinical sample, underscoring that autonomic dysregulation can be detected even in individuals without a diagnosed chronic pain condition. This highlights the biological plausibility of trauma-related autonomic alterations as early vulnerability markers that may precede the onset of persistent pain. Similar evidence has shown that reduced heart rate variability and heightened autonomic symptoms in trauma-exposed individuals are associated with increased risk for later somatic complaints, even in the absence of the current pathology e.g., [17,55]. Recognizing autonomic dysregulation in asymptomatic or subclinical populations may therefore provide an opportunity for early intervention and prevention by targeting physiological regulation before pain syndromes become chronic.

### 4.6. Limitations, Methodological Considerations, and Future Directions

Despite the strengths of the current study—including a moderately sized sample, validated instruments, and the use of structural equation modeling—several limitations should be acknowledged.

First, the cross-sectional design limits the ability to infer causal or temporal relationships among childhood trauma, autonomic dysregulation, and pain severity. Although the mediation model is statistically consistent with the proposed pathway, longitudinal studies are necessary to determine whether autonomic dysregulation temporally precedes the development or maintenance of chronic pain in trauma-exposed individuals (e.g., [106]). Experimental or prospective cohort designs could help clarify whether dysautonomia functions as a risk marker or as a consequence of pain-related distress. Importantly, while the statistical mediation model tested is consistent with the hypothesized pathway, it does not establish causal mediation. Statistical mediation in cross-sectional data reflects associations that are compatible with, but not definitive evidence of, temporal sequencing or causal influence. Longitudinal and experimental designs are required to confirm whether autonomic dysregulation temporally precedes and mechanistically mediates the link between childhood trauma and chronic pain outcomes.

Second, autonomic functioning was assessed using the Body Perception Questionnaire (BPQ), a validated self-report tool designed to capture subjective interoceptive awareness and perceived autonomic reactivity [39,69]. While practical in both clinical and community settings, self-report data may not align with objective physiological indicators. Previous studies have documented only moderate correlations between subjective and physiological measures of autonomic activity [107,108]. Future research should incorporate biophysiological measures such as heart rate variability (HRV), respiratory sinus arrhythmia, electrodermal activity, or baroreflex sensitivity to strengthen construct and ecological validity [15,109].

Similarly, pain severity was assessed using the Brief Pain Inventory (BPI), a widely validated and internationally used measure [44,110]. Although the BPI demonstrates strong psychometric properties and clinical relevance across both cancer and non-cancer pain, it is nonetheless a self-report instrument and may be influenced by recall bias, mood state, or context of assessment. This reliance on self-report for both autonomic and pain measures increases the potential for common-method variance and should be complemented by multimethod approaches in future studies. This limitation underscores the need to combine standardized questionnaires with ecological momentary assessment or objective functional measures (e.g., actigraphy and clinical pain thresholds) in future research. Additionally, potential confounding variables such as socioeconomic status, comorbid medical conditions, and lifestyle factors (e.g., physical activity, sleep, or substance use) were not systematically assessed. Also, environmental variables such as climate have also been shown to influence musculoskeletal pain trajectories in primary care populations [111]. Future studies should therefore integrate biomedical, psychological, and contextual determinants to better capture the complexity of pain outcomes. Their omission may limit the generalizability of findings. Additionally, the exclusion of the childhood sexual abuse (CSA) subscale due to floor effects should be acknowledged. Although this decision does not compromise the validity of the mediation findings, as the mechanistic focus on autonomic pathways remains theoretically and empirically supported, it limits the generalizability of the results to physical and emotional abuse. Future studies with greater variability in CSA exposure are needed to examine whether similar mechanisms apply across trauma subtypes.

Third, our global index of autonomic dysregulation may obscure clinically meaningful distinctions among autonomic subcomponents. Emerging evidence supports the idea that parasympathetic withdrawal, sympathetic overactivation, and separations between supradiaphragmatic and subdiaphragmatic autonomic symptoms are associated with different somatic outcomes. For example, subdiaphragmatic symptoms are linked to gastrointestinal distress, while supradiaphragmatic symptoms often reflect cardiorespiratory dysregulation [30]. Furthermore, interoceptive dysfunction—including reduced interoceptive accuracy and heightened perceptual bias—has been implicated in the development of somatic symptom disorders and anxiety-related psychopathology [64,112]. Disaggregating these autonomic dimensions may thus improve symptom specificity in future models and support the development of tailored interventions that target distinct autonomic and interoceptive mechanisms.

Moreover, although this study emphasized physiological mediators, chronic pain is a multidetermined phenomenon. Interactions between autonomic reactivity and cognitive–affective variables—such as pain catastrophizing, dissociation, or hypervigilance—as well as social contextual moderators, including socioeconomic stress and healthcare access, are theorized to shape symptom trajectories [113,114]. A more integrative transdiagnostic framework could improve the prediction of outcomes and facilitate the design of precision interventions. Finally, this study was conducted in Portugal, and cultural or contextual characteristics may limit the generalizability of findings to other populations. Replication in diverse international samples is therefore warranted.

Additionally, future studies should identify which autonomic domains are most relevant for predicting pain risk in trauma-exposed populations. For example, it remains unclear whether reduced vagal tone [115,116], enhanced cardiovascular reactivity [117], or visceral hypersensitivity [118] is the primary driver of chronic pain in these individuals. Clarifying these mechanisms would enhance our understanding of biopsychophysiological risk profiles and refine clinical tools for trauma-informed care in both mental health and primary care contexts.

Finally, the possibility of self-selection bias should be acknowledged. Participation required individuals to disclose sensitive information regarding childhood trauma, which may have discouraged those experiencing higher levels of distress, stigma, or avoidance. As a result, the trauma-exposed group may not fully represent the broader population of individuals with adverse experiences. This limitation could lead to an underestimation of trauma-related symptom severity or restricted variability in outcomes. Future studies should consider strategies to minimize self-selection effects, such as anonymous data collection, multimethod recruitment, or validation across population-based cohorts, to strengthen the representativeness and external validity of findings.

## 5. Conclusions

In conclusion, this study demonstrated that autonomic dysregulation mediates the association between childhood trauma and pain severity. Rather than supporting a direct trauma–pain association, the findings underscore autonomic reactivity as a critical physiological pathway through which early adversity confers long-term somatic vulnerability. These results highlight the need to account for stress-regulatory processes in the investigation of trauma-related health outcomes and suggest that interventions targeting autonomic balance may play a pivotal role in reducing pain susceptibility among trauma-exposed individuals. By elucidating the embodied effects of early adversity, this work advances a more refined biopsychosocial model of chronic pain and informs the development of integrated trauma-informed healthcare strategies. Future research should employ prospective, longitudinal, and cross-cultural designs to validate the mediation pathway identified here and to clarify its generalizability across diverse populations and healthcare contexts.

## Figures and Tables

**Table 1:** Descriptive statistics and group comparisons (trauma-exposed group vs. comparison group).

Variable	Overall (n = 124)	Trauma-Exposed Group (n = 64)	Comparison Group (n = 60)	Statistical Test (t/χ^2^)	*p*-Value	Cohen’s *d* [95% CI]
Age (M ± SD)	37.45 (13.48)	42.78 (14.05)	31.77 (10.21)	t(122) = −4.97	<0.001 ***	0.89 [0.52, 1.26]
Gender (% Female)	66.9%	70.3%	63.3%	χ^2^(1) = 0.682	0.409	—
CTQ—Childhood Physical and Emotional Abuse (CPEA)	7.96(3.49)	8.88 (4.13)	6.99 (2.30)	t(122) = −3.12	0.002 **	0.56 [0.20, 0.92]
LEC-17—Cumulative Trauma Exposure	10.41 (8.10)	13.20 (8.43)	7.43 (6.60)	t(122) = −4.23	<0.001 ***	0.76 [0.39, 1.12]
BPQ—Autonomic Reactivity	33.88 (14.67)	38.19 (18.26)	29.28 (7.13)	t(122)= −3.53	<0.001 ***	0.64 [0.27, 1.00]
Pain Severity (Brief Pain Inventory)	7.69(8.58)	7.03 (9.36)	8.40 (7.68)	t(122) = 0,89	0.377	−0.16 [−0.51, 0.19]

Note: Values are presented as M (SD) for continuous variables and % for categorical variables. Independent sample *t*-tests were used for continuous variables, and χ^2^ tests were used for categorical variables. Cohen’s *d* with 95% confidence intervals is reported for continuous variables, indicating the effect size magnitude (0.20 = small, 0.50 = medium, and 0.80 = large). *p* < 0.01 **, *p* < 0.001 ***.

**Table 2:** Pearson correlations between childhood trauma, autonomic reactivity, pain severity, age, and gender.

	CTQ–CPEA	BPQ—Reactivity	Pain Severity	Age	Gender
CTQ–CPEA	—	0.524 ***	0.120	0.136	−0.105
BPQ—Reactivity		—	0.408 ***	0.206 *	−0.194 *
Pain Severity			—	0.081	−0.208 *
Age				—	0.000
Gender					—

Note: Pearson correlation coefficients (r) are presented. Gender was coded as 0 = female, 1 = male. Also, 95% confidence intervals for correlations are available upon request; the pattern of significance remained unchanged. *p* < 0.05 *, *p* < 0.001 ***.

**Table 3:** Hierarchical regression models predict autonomic reactivity and pain severity from demographic and trauma-related variables.

Regression Step	Predictor	Outcome	B (unstd.) [95% CI]	β (std.)	*p*-value	R^2^	F(df)
Regression 1: Predicting Autonomic Reactivity (BPQ)						0.41	F(4,119) = 20.65, *p* < 0.001
	Age	BPQ	0.123 [−0.031, 0.277]	0.113	0.116		
	Gender	BPQ	−3.854 [−8.215, 0.508]	−0.124	0.083		
	LEC-17	BPQ	0.609 [0.335, 0.882]	0.336	<0.001 ***		
	CPEA	BPQ	1.558 [0.921, 2.194]	0.370	<0.001 ***		
Regression 2: Predicting Pain Severity (CPEA only)						0.05	F(2,121) = 3.37, *p* = 0.038
	Gender	Pain Severity	−3.58 [−6.778, −0.380]	−0.20	0.029 *		
	CPEA	Pain Severity	0.24 [−0.189, 0.677]	0.10	0.267		
Regression 3: Predicting Pain Severity (with BPQ)						0.18	F(2,121) = 13.59, *p* < 0.001
	Gender	Pain Severity	−2.43 [−5.437, 0.585]	−0.13	0.0113		
	BPQ	Pain Severity	0.22 [0.126, 0.320]	0.38	<0.001 ***		

Note: BPQ = Body Perception Questionnaire (autonomic reactivity score); CPEA = Childhood physical and emotional abuse subscale (from the CTQ); LEC-17 = Life Events Checklist (cumulative trauma exposure); and Pain Severity = Brief Pain Inventory severity score. Unstandardized coefficients (B) are presented with 95% confidence intervals. Standardized coefficients (β) are also reported. R^2^ refers to the proportion of variance explained by the full model. F(df) reflects model-level significance. Gender was coded as 0 = female, 1 = male. *p* < 0.05 *, *p* < 0.001 ***.

**Table 4:** Standardized mediation effects of childhood trauma (CTQ_CPEA) on pain severity via autonomic reactivity (BPQ).

Pathway	Total Effect β [95% CI]	Direct Effect β [95% CI]	Indirect Effect β [95% CI]
CTQ_CPEA → BPQ → Pain Severity	0.090 [−0.198, 0.397]	−0.130 [−0.379, 0.131]	0.220 * [0.087, 0.422]

Note: β = standardized coefficient. CI = confidence interval (bias-corrected). BPQ = Body Perception Questionnaire—autonomic reactivity total score. CTQ_CPEA = Childhood Trauma Questionnaire—physical and emotional abuse. Pain severity measured by BPI. *p* < 0.05 *.

**Table 5:** Parameter invariance test by gender.

Constraint	Path Compared	χ^2^	df	*p*-Value
p_1_ = p_10_	a: CTQ_CPEA → BPQ	3.880	1	0.049
p_2_ = p_11_	b: BPQ → Pain	0.005	1	0.941
p_3_ = p_12_	c: CTQ_CPEA → Pain	0.361	1	0.548

Note: Score tests indicate parameter invariance across gender groups (*p* > 0.05), except for path a (CTQ_CPEA → BPQ), which shows a marginally significant difference (*p* = 0.049).

**Table 6:** Standardized coefficients in the gender-weighted model.

Group	Path	Estimate (Std.all)	95% CI	*p*-Value
Men	a: CTQ_CPEA → BPQ	0.427	[0.243, 0.611]	<0.001 ***
Men	b: BPQ → Pain	0.391	[0.266, 0.516]	<0.001 ***
Men	c: CTQ_CPEA → Pain	−0.098	[−0.717, 0.521]	0.315
Women	a: CTQ_CPEA → BPQ	0.523	[0.298, 0.748]	<0.001 ***
Women	b: BPQ → Pain	0.470	[0.345, 0.595]	<0.001 ***
Women	c: CTQ_CPEA → Pain	−0.144	[−0.763, 0.475]	0.315

Note: Results from the gender-weighted SEM (83 women; 41 men) support the robustness of the mediation effects. The trauma–pain association appears to be similarly mediated by autonomic reactivity across genders. The direct path (c) was not significant in either group, reinforcing the presence of an indirect effect. All coefficients are standardized (Std.all), indicating effect sizes expressed in standard deviation units. Corresponding 95% confidence intervals are reported to facilitate interpretation of precision. *p* < 0.001 ***.

## Data Availability

The data supporting the findings of this study are not publicly available due to ethical and privacy restrictions but may be made available by the corresponding author upon reasonable request and with appropriate institutional approvals.

## Abbreviations

The following abbreviations are used in this manuscript:

ANS	Autonomic Nervous System
BPI	Brief Pain Inventory
BPQ	Body Perception Questionnaire
CI	Confidence Interval
CPEA	Childhood Physical and Emotional Abuse
CSA	Childhood Sexual Abuse
CTQ	Childhood Trauma Questionnaire
GNR	Guarda Nacional Republicana
HRV	Heart Rate Variability
LEC-17	Life Events Checklist for DSM-IV (17 items)
PTSD	Post-Traumatic Stress Disorder
SEM	Structural Equation Modeling
VNS	Vagus Nerve Stimulation
